# Immediate Dental Implant Placement in the Oncologic Setting: A Conceptual Framework

**DOI:** 10.1097/GOX.0000000000003671

**Published:** 2021-09-17

**Authors:** Rachel A. Anolik, Jonas A. Nelson, Evan B. Rosen, Joseph Disa, Evan Matros, Robert J. Allen

**Affiliations:** From the * Division of Plastic and Reconstructive Surgery, Department of General Surgery, Memorial Sloan Kettering Cancer Center, New York, N.Y.; † Miami Cancer Institute, Miami, Fla.

## Abstract

Historically, immediate dental implants have been reserved for patients with benign disease, with full dental rehabilitation rarely being accomplished in the oncologic setting due to concerns related to implant survival, flap compromise, and delay in initiation of adjuvant therapy. Recent developments in technology have made immediate dental implants using virtual surgical planning safe and reliable. At Memorial Sloan Kettering Cancer Center, we have implemented a workflow for immediate dental implant placement in the oncologic patient population that has become a routine part of maxillary and mandibular reconstruction. This approach begins with a multidisciplinary virtual surgical planning session and custom dental splints to be used for cutting and inset guides. Dental implants are placed intraoperatively at the time of tumor resection and reconstruction with the fibula flap. A temporary prosthesis, which can be worn during radiation therapy, is placed following a vestibuloplasty, approximately 4–6 weeks after the initial reconstruction. After the completion of radiation therapy and the resolution of edema, a permanent prosthesis is placed. When critically evaluating our experience, we have found that patients undergoing immediate dental implant placement have higher rates of implant survival and no delay in adjuvant therapy. The protocol described here in detail has successfully expanded the indications for immediate dental rehabilitation in the oncologic patient population.

## INTRODUCTION

Oncologic maxillomandibular reconstruction aims to restore form, function, and aesthetics following tumor ablation. This is most commonly performed with the free fibula flap as first described by Hidalgo in 1989.^1^ Because the fibula flap allows for both bony and soft tissue reconstruction, it has become the workhorse for mandible reconstruction to restore facial contour/height, mastication, and physical appearance in the oncologic patient.^2^ Historically, full functional dental rehabilitation has rarely been achieved in this patient population for reasons including, but not limited to fear of devascularization to the fibula, unpredictable soft tissue requirements dictated by oncologic needs, the inability to accurately and precisely place dental implants in the fibula graft, fear of delaying adjuvant therapy, instigation of osteoradionecrosis, the presence of trismus precluding mastication, and perceived high patient mortality related to disease.^3–6^ For the select few patients who underwent delayed dental implant placement, the process was typically drawn out over several years in multiple stages.^7^ These concerns have resulted in a failure of full functional reconstruction in the majority of our oncologic maxillomandibular reconstruction patients.

Recent technological advances have begun to change the scenario for some patients undergoing maxillary or mandibular reconstruction. This is best observed in the groundbreaking “jaw-in-a-day” procedure described by Levine et al in which virtual surgical planning (VSP) is used in conjunction with computer-aided design and computer-aided manufacturing to provide full functional maxillomandibular reconstruction, including dental restoration in one surgery.^8^ While reserved almost exclusively for benign tumors of the mandible or maxilla (eg, ameloblastoma), this procedure calls for immediate dental implantation into the fibula flap and placement of a temporary dental prosthesis. When the patient awakes from the procedure, they not only have a reconstructed jaw, but their dentition has been restored and occlusion maintained. In contrast, the oncologic patient has two concerns that need to be considered and overcome to make it a reality. These include the need for soft tissue resurfacing of the oral cavity following tumor removal and the ability to accommodate timely adjuvant radiation.

With this proof of concept in mind, we recently began to consider its application in oncologic patients at Memorial Sloan Kettering Cancer Center. To achieve our goals of increased numbers of patients undergoing full functional dental rehabilitation and decreasing the time that this process took, a systematic review of the literature was performed to critically evaluate the aforementioned concerns. Forty-two studies were ultimately included, showing promising results in support of immediate dental rehabilitation despite long-held reservations. While overall survival was significantly higher in the nonirradiated dental implant group (implant survival of 95% at an average of 37 months follow-up), there was a significant difference seen within the patients undergoing radiation therapy, specifically as it related to the timing of implant placement and radiation. The results demonstrated that dental implant survival in patients who underwent flap irradiation before implant placement was 81%, with an average follow up of 45 months. However, patients who received dental implants before radiation therapy had an overall implant survival of 88% with an average follow up of 30 months. Essentially, implant survival was significantly improved when placed into the fibula graft before radiation therapy when compared with those placed in the fibula graft following radiation therapy (88 versus 81%, *P* = 0.01).^9^ The underlying premise here is that patients have between 4 and 6 weeks to allow the implant to osseointegrate before radiation initiation. This review also found a trend toward improved quality of life in patients who underwent dental rehabilitation, which may be attributed to both aesthetic and functional outcomes.^10, 11^ Collectively and after this comprehensive review of the literature, we determined that immediate dental implant placement (IDIP) showed the most promise in applying full functional dental rehabilitation in oncologic maxillomandibular reconstruction.

## TECHNIQUE

Once we established that immediate dental implants were a reasonable option supported by the literature, a protocol was designed to put into use at Memorial Sloan Kettering Cancer Center. Our goal was to improve patient outcomes and quality of life by providing IDIP in our patient population. In conjunction with the head and neck oncologist, dental oncologist and plastic surgeons, we developed the following planning and surgical workflow. (**See table, Supplemental Digital Content 1,** which displays the workflow and timeline for immediate dental rehabilitation in oncologic osseous jaw reconstruction. CAD-CAM: computer-aided design-computer-aided manufacturing; PRS: plastic and reconstructive surgeon. **http://links.lww.com/PRSGO/B738**.)

### Virtual Surgical Planning

Six days to 2 weeks preoperatively, the reconstructive, ablative, and dental oncology teams participate in an online VSP meeting facilitated by a media technician. The purpose of this multidisciplinary meeting was to bring together the different disciplines and create a unified operative plan to maximize efficiency in the operating room.^12, 13^ In preparation for this meeting, the patient undergoes a computed topography mandible or maxilla and a computed topographic angiography of the lower extremities. These studies are uploaded for evaluation and used to perform the virtual surgery online. The mandibular osteotomies and resultant defect are planned by the ablative team. To ensure negative margins, the ablative surgeons are encouraged to go wide with the planned resection. (Alternatively, a narrow and a wide plan may be devised; however, this adds to the cost of the reconstruction.) Mandibular cutting guides can then be designed for use intraoperatively.

The reconstructive surgeon and dental oncologist will then choose a segment of fibula to be harvested along with the location of dental implant placement based on fibula size and shape as well as perforator location. (Of note, a double barrel fibula flap is not performed in the oncologic setting due to the frequent need for soft tissue reconstruction. We have found that a double barrel fibula flap creates excess height of the reconstruction, which can either interfere with dental prosthesis placement or result in an open bite when placing the prosthesis at the time of vestibuloplasty.) The size and shape of the fibula is evaluated to select the appropriate location for dental implant placement. The implant must engage both cortices of the fibula with 1 mm of surrounding cortical bone. As the shape of the fibula changes not only between patients but also within the same patient, some areas of the fibula will provide a more favorable implant location than others. The precise placement of implants afforded by VSP is essential in preventing buccal or lingual rotation of the implant and maintaining adequate occlusion once full dental restoration has occurred.^14^ These data also determine the size and length of the dental implants, decreasing the risk of iatrogenic fracture of the fibula during placement. Once the fibular osteotomies and location of the dental implants have been planned, a custom reconstruction bar can be designed for manufacture (this usually requires 10–14 days to prepare) or mini-plates can be contoured intraoperatively. The detail provided by VSP also allows for preoperative prediction on number, location, and length of screws to be used during rigid fixation of the fibula and of the fibula to the native mandible.

Finally, the computed topographic angiography can be used to visualize the peroneal artery perforator location, aiding in soft tissue planning as well as a reference point for accurate placement of the fibula cutting guide intraoperatively. Other information that can be gathered from the computed topographic angiography include the pedicle length, determined by the tibial-peroneal artery bifurcation, or the presence of vascular anomalies that may preclude the use of the fibula for reconstruction. Ultimately, it is the VSP technology that enables the multidisciplinary team to accurately and precisely provide full dental rehabilitation to the oncologic patient, and even small alterations to the plan intraoperatively can result in malocclusion or an inability to provide full dental rehabilitation.^15^

### Evolution of Occlusion-based Cutting and Inset Guides

**Fig. 1.**
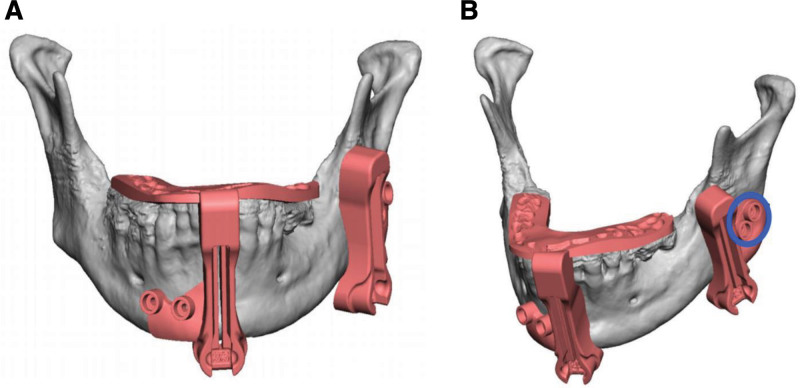
Occlusion-based cutting guide. Anterior-posterior (A) and oblique view (B) of VSP rendering of the occlusion-based cutting guide for anterior osteotomy and nonocclusion-based cutting guide of posterior osteotomy.

Traditionally, maxillofacial cutting guides have been fashioned using the bony landmarks of the facial skeleton (foramina, bony prominences, and contour of the lower border of the mandible); however, we have noted some limitations and adjusted our approach to designing osteotomy cutting guides. Using bony landmarks as a guide, we frequently noted small discrepancies in bony apposition and occlusion following rigid fixation. We hypothesized that the discrepancies resulted from slight movement of the cutting guides caused by the muscles of mastication following complete osteotomy during tumor ablation. To decrease the chances that this occurred, we began performing only unicortical osteotomies at each site before completing the osteotomy at each location, noting improvement in the ultimate apposition of the fibula to the native mandible. While the apposition was improved, we still noted small changes in buccal or lingual rotation of the implants. Even with small amounts of rotation, malocclusion can result following placement of the dental prosthesis; so custom-fabricated cutting and inset guides are now fashioned with the assistance of the dental surgery team. To create these guides, a digital 3D model of the patient’s occlusion must be obtained using an intraoral scanner or dental molds. (Computed topography data are often not sufficient due to dental artifact.) The guide has four different registration areas on both the maxillary and mandibular dentition that allows for reliable placement in the oral cavity (Fig. 1). The patient must be placed in full occlusion for placement of both the cutting guides and subsequently for orientation of the fibula flap in the defect (inset guide). Using occlusal landmarks as opposed to bony landmarks on the remnant mandible has lead to more accurate osteotomies and consistent inset of the fibula flap due to the increased points of reference.^14^ This is of utmost importance when placing dental implants to ensure functionality.

### Intraoperative Workflow

Tumor extirpation is performed by the head and neck surgeon with assistance from the plastic surgeon when making the mandibular osteotomies. The aforementioned cutting guides from VSP are used, and special attention is paid to the lower border of the mandible. If predictive fixation holes are utilized, these should be drilled in the native mandible before the osteotomies being performed. The accuracy of this cut is crucial to achieve good bony contact between the native mandible and the fibula when performing rigid fixation. Once the specimen has been removed, the margins are then sent for frozen examination if deemed necessary by the ablative surgeon.

**Fig. 2.**
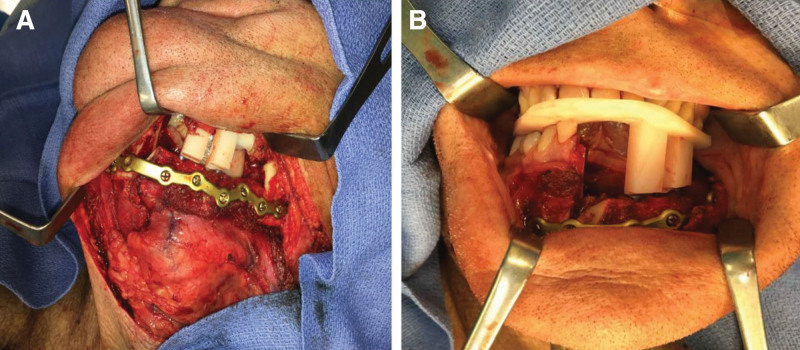
Occlusal-based inset guide and rigid fixation. Rigid fixation of fibula flap to native mandible with immediate dental implants and occlusion-based inset guide seen from neck incision (A) and intraoral view (B). The prefabricated occlusal splint is used to guide the inset of the fibula flap and helps prevents buccal or lingual rotation of the implants.

The fibula flap is simultaneously elevated by the reconstructive team. Once the osseous or osteocutaneous flap has been isolated on its pedicle, the fibula cutting guide is brought in and location confirmed on the fibula. Precise placement of the guide is key as subtle differences in the size and shape of the fibula will cause differences in occlusion. We have found the preoperative location of the perforator to be the easiest and most reliable point of reference for fibula cutting guide placement. With the guide attached to the fibula, a needle point bovie is used to mark the planned osteotomy and implant locations. The cutting guide is then removed, and the periosteum is cleared in these areas. Depending on the shape of the fibula, a small amount of the bone may need to be shaved down to allow for a flat surface for implant placement. The fibula cutting guide is then placed back on the fibula, and the dental oncologist places the dental implants into the bone. Once the margins return negative, predictive fixation holes are drilled in the fibula, the osteotomies are made, and the cutting guide may then be removed. Rigid fixation can be performed with the flap still attached to the leg, decreasing the potential ischemia time to the flap. (Rigid fixation of the fibula can also be performed after harvest of the flap, depending on surgeon preference.) Next, the peroneal vessels are ligated and the fibula flap is brought to the mandible. The aforementioned prefabricated occlusal splint is used to guide the inset of the fibula flap with the goal of preventing buccal or lingual rotation of the implants, which impacts future prosthesis placement. (The implants may accommodate up to 30 degrees of correction with the final prosthesis.) The fibula is fixated to the native mandible using the occlusion-based inset guides. Mini-plates or a custom reconstruction plate can be utilized for fixation, with or without predrilled, predictive fixation holes. The skin paddle is then brought over the construct and into the oral defect for inset. Microvascular anastomoses are then performed in a standard fashion. Rigid fixation is then performed with the aid of previously mentioned occlusion-based guides, which can be done using prefabricated mini-plates or reconstruction bars, in accordance with surgeon preference (Fig. 2).

### Postoperative Course

**Fig. 3.**
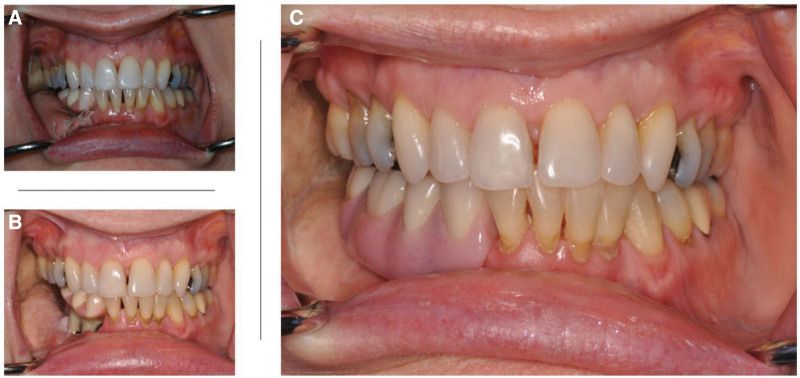
Final dental prosthesis. A, Temporary dental prosthesis in place after vestibuloplasty, approximately 6 weeks after reconstruction. B, Temporary dental prosthesis after radiation-induced shrinkage of the flap. C, Final prosthesis in place after completion of radiation therapy.

There is no change to the immediate postoperative protocol for IDIP patients compared with those without IDIP while in the hospital. Assuming a routine postoperative course, vestibuloplasty is planned approximately 4–6 weeks following the initial surgery. The 4–6 week time frame is chosen based on the current implant protocols with newer generation implants that have been shown to osseointegrate within 4 weeks of implantation.^16^ The range in time presents the variable recovery patterns of our patients as well as the expected timing of or even need for adjuvant radiation therapy (For those patients that do not require adjuvant XRT, the length of time between initial surgery and vestibuloplasty can be delayed based on patient preference). During the vestibuloplasty, the reconstructive surgeon accesses the abutments percutaneously and provides exposure for the dental surgery team. The abutments are then exchanged for definitive implants with sterile protective caps. The plastic surgeon then closes skin around the base of the abutments. Any revisions or removal of excess flap skin may also be done at this time. Placement of the dental prosthesis is performed in the dental clinic 1–3 days following vestibuloplasty. If radiation is indicated, this occurs 6–8 weeks after the initial surgery, giving the fibula flap and the dental implants adequate time to heal. The temporary prosthesis is used throughout radiation therapy, negating the need for bulky intraoral bolsters more commonly used during this therapy. The prosthesis bolsters the gingivobuccal sulcus and maintains this space throughout treatment, limiting the potential radiation-induced constriction to this area. Not only does the prosthesis maintain the gingivobuccal sulcus and add another layer of fixation to the reconstruction, but patients are also more likely to continue with intraoral exercises and mastication throughout the radiation process with it in place. This unforeseen benefit may limit trismus and improve quality of life.^17^ It is also reasonable to assume that continued resistance as is seen with mastication may also aid in bony remodeling and strength.^18^ After the resolution of radiation-induced edema, a temporary prosthesis may be exchanged for a permanent one in the dental oncology clinic. This usually occurs around 6 months after the completion of radiation (Fig. 3).

### Outcomes

After establishing this workflow, we sought to understand the early outcomes and complications as a quality assurance measure. ^19^ To ensure the short term (<90 days) safety and efficacy of this workflow, we designed a noninferiority study comparing similar cohorts of patients that either underwent IDIP or did not (historical cohort). Examining a 16-month period from May 2017 through August of 2018, 27 patients underwent oncologic jaw reconstruction with IDIP (72 total implants placed). This cohort of patients was compared to a historical cohort of 34 patients that underwent reconstruction without IDIP. The primary outcomes of concern were time to radiation therapy, number of patients completing dental rehabilitation, time to dental rehabilitation and implant survival. The results of this study found no significant change in early complications (minor or major), no significant delay in adjuvant therapies and a significant increase in the number of patients completing total dental rehabilitation (51.8% versus 0.0%, *P* < 0.001). The study concluded that IDIP in oncologic jaw reconstruction is safe, does not delay necessary adjuvant therapies, increases the number of patients completing full functional dental rehabilitation, and decreases the time required to do so.^20^

## DISCUSSION

Immediate dental implant placement in maxillomandibular reconstruction was first described by Urken in 1998.^2^ Since that time, technological advancements in imaging, VSP and computer-aided design and computer-aided manufacturing have improved our ability to accurately plan and execute this for our patients, resulting in the concept of “jaw-in-a-day”.^8^ Despite these advances, however, there has been resistance in applying this technique to the oncologic setting. For fear of delaying adjuvant therapies, the theoretical effects that this could have on healing of the fibula graft with IDIP, as well as the variable soft tissue requirements for these patients, many oncologic patients never achieve full dental rehabilitation. However, these fears are not supported by the literature. To the contrary, our systematic review of the literature concluded that dental implants have a high survival rate in fibula grafts, and, more importantly, that there was a greater percentage of implant survival when placed before radiation.^9^ Based on these preliminary studies, we developed a novel workflow for IDIP in oncologic maxillomandibular reconstruction as described above.

In May of 2017, Memorial Sloan Kettering Cancer Center instituted the novel IDIP workflow in oncologic patients. The results of the short-term, noninferiority results suggest that this workflow does not increase patient complications or time to adjuvant radiation, but does significant improve chances of patients achieving dental restoration. Certainly, complications still occur, and not all patients who undergo IDIP have postoperative courses which enable vestibuloplasty before radiation. However, even these patients have the foundation for dental restoration. Currently, all patients eligible for bony reconstruction of the jaw are candidates for IDIP at our institution; however, we do not place implants distal to the first premolar because of difficulties in exposing these implants at the time of vestibuloplasty. Thus, a bony reconstruction posterior to the first premolar is the only relative contraindication to providing this service.

Throughout this process, our group has instituted several modifications to improve the accuracy and precision of IDIP and noted several unexpected benefits of this approach. First, using bony landmarks for the design and placement of cutting guides is inferior to more recent occlusion-based guides.^11, 14^ Occlusion-based guides, when possible, provide for many more points of reference when placing the cutting guides, ensuring that osteotomies are accurate. This improves bony apposition once the fibula graft is rigidly fixated to the mandible. Additionally, occlusion-based inset guides can also be used to limit either buccal or lingual angulation of the dental implants on inset of the graft. With this modification, we have found improved postoperative occlusion upon placement of the dental prosthesis. Secondly, the temporary dental prosthesis that is placed before the initiation of radiation therapy is an excellent substitute for the bulky intraoral molds normally used to protect the gingivobuccal sulcus during radiation therapy. While further studies are needed to determine the long-term effects that the temporary prosthesis has on the effects of radiation-induced fibrosis and post-therapy trismus, our initial observations have been promising.

The ever-evolving technology and knowledge that make IDIP possible is sure to improve our reconstructive results in the oncologic patient. Further studies are planned and include not only long-term results of IDIP but also a cost-based analysis of this workflow. Additionally, quality of life and patient-reported outcome studies are needed to validate what we believe to be inevitable: full functional dental rehabilitation will improve patients’ quality of life. Based on our initial outcomes following institution of a novel workflow for IDIP in oncologic jaw reconstruction, we feel that it is not only safe but also should become the standard of care in this patient population.

## Supplementary Material


