# Supplementary figures and images for: Immediate Dental Implant Placement in the Oncologic Setting: A Conceptual Framework
Source: Plast Reconstr Surg Glob Open. 2021 Sep 17;9(9):e3671. doi: 10.1097/GOX.0000000000003671 (PMC8447991; doi:10.1097/GOX.0000000000003671)

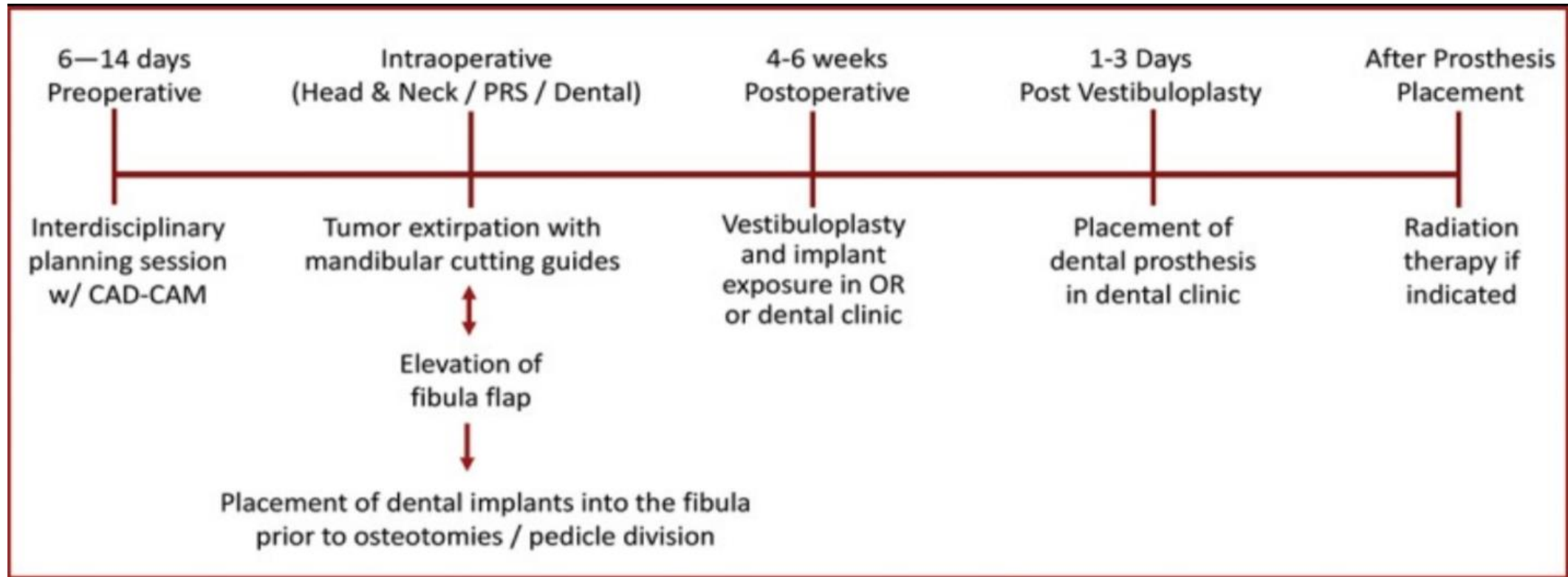

Supplement: Supplementary file 1 [file gox-9-e3671-s001.pdf]
